# Removing Dust From the German Health Care System by Introducing Health Apps Into Standard Care: Semistructured Interview Study

**DOI:** 10.2196/42186

**Published:** 2023-05-04

**Authors:** Alexandra Heidel, Christian Hagist, Stefan Spinler, Michael Schoeneberger

**Affiliations:** 1 WHU - Otto Beisheim School of Management Vallendar Germany

**Keywords:** health apps, DVG, Digitale Versorgung Gesetz, Digital Healthcare Act, mobile health, mHealth, German statutory health care system, interview study

## Abstract

**Background:**

In 2019, Germany launched the Digital Healthcare Act. The reform enables physicians to prescribe health apps as treatments to their statutory-insured patients.

**Objective:**

We aimed to determine the extent to which the integration of health apps into standard care could be considered beneficial and which aspects of the regulation could still be improved.

**Methods:**

We conducted a semistructured interview study with 23 stakeholders in Germany and analyzed them thematically. We used descriptive coding for the first-order codes and pattern coding for the second-order codes.

**Results:**

We created 79 first-order codes and 9 second-order codes following the interview study. Most stakeholders argued that the option of prescribing health apps could improve treatment quality.

**Conclusions:**

The inclusion of health apps into German standard care could improve the quality of treatment by expanding treatment portfolios. The educational elements of the apps might additionally lead to more patient emancipation through a better understanding of personal conditions. Location and time flexibility are the biggest advantages of the new technologies, but they also raise the most significant concerns for stakeholders because app use requires personal initiative and self-motivation. Overall, stakeholders agree that the Digital Healthcare Act has the potential to remove dust from the German health care system.

## Introduction

### Overview

In 2019, Germany was the first country worldwide to launch an act that enabled medical doctors to prescribe health apps as treatments to their patients—the so-called Digital Healthcare Act (Digitale Versorgung Gesetz [DVG]) [1]. Therefore, health apps became part of the German standard health benefit basket, financed by the statutory sickness funds. Previously, health apps were offered on a voluntary and discretionary basis in Germany, depending on the decisions of individual sickness funds or private health insurance companies. The statutory health care system in general was not covering the costs of any health app. Now, health apps can enter a preceding certification process by the “Federal Institute for Drugs and Medical Devices” (Bundesinstitut für Arzneimittel und Medizinprodukte [BfArM]). If the certification process is successful, the health app becomes a so-called “digital health app” (Digitale Gesundheitsanwendung [DiGA]).

The validation and certification process for these health apps is an entirely new process and still leaves room for future research and discussion [2]. The Digital Healthcare Act has the potential to decrease the costs associated with unnecessary doctor’s visits and substitute or complement other traditional treatments through digital initiatives related to patient education and self-management [3].

The BfArM has received 161 applications for admission to the DiGA index by January 2023, which would sanction these apps as prescribable treatments [4]. In January 2023, already 40 health apps were listed in the DiGA index, and they are now available via a physician’s prescription [5]. Many countries, especially in Europe, are observing the DiGA development in Germany closely, as they aim to introduce similar reimbursement strategies to disburden the health care system and increase the level of digitization of standard care. Belgium and France identified as following the German DiGA reimbursement example [6].

### Certification Process of DiGA

The certification process for health apps and digital health devices was specified within the digital device regulation (Digitale-Gesundheitsanwendungen-Verordnung [DiGAV]) [7]. The BfArM published guidelines for health apps based on § 139e clause 8 (1) German social code (Sozialgesetzbuch [SGB V]) [8]. The guidelines highlight that DiGA need to be medical devices of the risk classes I or IIa, according to Eropean Union regulation 2017/745 [9]. The guidelines explain the procedure for admission to the DiGA index. First, the app provider needs to apply to the BfArM to be admitted to the official index of reimbursable DiGA. The BfArM then examines the app or the digital health device for safety, quality, data security, data privacy, and several functional requirements within a 3-month period after the application was submitted. Thereafter, the BfArM conducts a first assessment of the potential positive treatment effects of the app. If this evidence is not yet sufficiently demonstrated in studies and publications but all other requirements are fulfilled, the health app may still receive preliminary acceptance to the index according to § 139e SGB V [2]. During this phase, the health app is in a 12-month test phase.

The app can be prescribed through medical doctors during the test phase, and the health app provider may set the price for market entry. After 12 months, the health app provider needs to demonstrate sufficient proof of positive care effects. The legislator used the term positive care effect in the DVG and defined the concept as a medical beneficial outcome or patient-relevant procedural improvement in care [1]. If sufficient proof of a positive care effect cannot be demonstrated, the app is removed from the index, and a prescription is no longer possible. If the health app provider has demonstrated sufficient effectiveness, the price for use of the app is negotiated with the national association of statutory health sickness funds [2]. This system of preliminary market access and reimbursement is supposed to facilitate innovation within the health care sector. After negotiating the final price, the app is permanently accepted to the DiGA index [2].

### The DiGA Prescription Process

The DVG is one of many initiatives by the German Federal Ministry of Health to modernize and digitize the German health care system. The aim of the act is to quickly introduce innovative digital treatment solutions into the standard care portfolio and to give statutory sickness funds the opportunity to encourage more efficiency and higher quality treatment [1]. The DVG enabled statutory health–insured patients to claim digital solutions, if available, for disease management and treatment. Physicians, as the gatekeepers of the German health care system, play a major role in the success of the DVG. According to the act, physicians are required to recommend and prescribe suitable health apps and supervise the app use of the patients according to their individual disease progression [1]. Compensation for this supervision is not yet sufficiently regulated. Hence, the reform contains a subsection stating that practitioners’ efforts shall be compensated, but a clear guideline and incentive system is yet to be negotiated [1].

In May 2020, the board of the German Medical Association recommended compensation for practitioners prescribing and providing advice upon first-time use of a specific DiGA, according to the billing code for practitioners (Gebührenordnungsposition [GOP]) as GOP 01470 [10]. This code reimburses the practitioner an amount of 2.00 € (US $2.21) and may only be billed once per app [10]. Just recently, a new billing code numbered 86700 has been introduced to reimburse practitioners to monitor twice a year the progress of the app use. However, not all medical specialist groups, such as urologists, were included in the compensation logic; they are, therefore, not allowed to use the billing code for supervision [11]. This is a symbolic starting point but might not be enough to set an effective incentive system for practitioners.

### The German Ambulatory Setting

In Germany, most physicians in the ambulatory sector are self-employed. Their motivation to enhance and recommend the use of health apps might also be debatable given the lack of financial incentives to do so. Many private practitioners lack a range of digital solutions in their practices [12]. Approximately, only 56% to 58% of German private practitioners have already digitized processes, such as patient documentation, appointment planning, and waiting time management, for their practices [12]. Just 37% of resident doctors are willing to standardize their patient documentation to accelerate the introduction of a digital patient file to encourage better patient data exchange between different specializations [12]. In Germany, there is an imbalance between the demand and supply of physicians, partly explained by a general shortage of physicians, especially in rural areas, and partly explained by the unique statutory health care system and the apparent nearly unlimited and free doctor’s treatment portfolio for statutory health–insured patients [13].

Conducting an interview study, we aimed to determine the extent to which the integration of health apps into standard care could be considered beneficial by different stakeholders and which aspects of the regulation could still be improved. We expected a general reticence toward the DVG from most of the stakeholders. Yet we also expected that the acceptance of app treatments is currently changing due to the experiences of the COVID-19 crisis since location-independent, flexible, and at-home practicable solutions have gained importance.

## Methods

### Procedure

We used an interview study approach to explore different aspects of the introduction of mobile health services in the German statutory health care system. We conducted a semistructured interview study with 23 stakeholders in Germany and thematically analyzed those interviews [14].

First, we identified relevant stakeholder groups to guide sampling. The stakeholder groups are the following:

- Certification institutions: institution that currently and in the past examined and certified medical devices, digital preventive care solutions, or DiGA.
- Medical doctors: physicians who work in the ambulatory sector in different specialties.
- Health app producers: companies that develop digital medical solutions.
- Statutory sickness funds representatives: representatives who work for statutory sickness funds within a DiGA business unit or project group.
- Political representatives: politicians who work for regional or federal ministries.
- Medical chamber representatives: representatives who work for different regional medical chambers, which are compulsory institutions that represent the interests of physicians in Germany.

We contacted 65 stakeholders via purposeful sampling based on their profession and expertise between October 2019 and December 2019 [15]. Thereafter, 23/65 stakeholders responded to and participated in the study. Second, we created a suitable interview guide (Multimedia Appendix 1), discussed, and tested the questions in a real interview scenario with a previously selected stakeholder. We conducted the interviews between October 2019 and January 2020 with a certification body representative (1/23), medical doctors (9/23), health app producers (2/23), medical chambers (4/23), political representatives (5/23), and statutory sickness funds’ representatives (2/23). Most interview partners were middle-aged (Table 1) and almost equally distributed by gender (13/23 were male and 10/23 were female).

We used the software ATLAS.ti (version 9.0.18; ATLAS.ti Scientific Software Development Gmb H) to thematically analyze and cluster the transcripts. Two researchers independently coded the transcripts using thematically relevant first- and second-order codes and found consensus about the final codes by merging the coding data, and therefore, consolidating the most important themes (final codes) through educated discussions [16]. The procedure to establish first-order codes consisted of highlighting the important parts of the transcripts and summarizing these through descriptive first-order codes [16-19]. In the second step, we aggregated the descriptive first-order codes so that all duplicates could be removed without any loss of important information. In the final step, we used pattern coding to organize and cluster the second-order codes by the most relevant topics [16,20].

**Table 1:** Descriptive statistics interview study.

Descriptive statistics	Distribution within sample					
	Gender, n/N (%)			Age group (years), n/N (%)		
	Female	Male	<35		35-50	>50
Medical doctors	4/9 (53)	5/9 (47)	2/9 (22)		4/9 (45)	3/9 (33)
Statutory sickness funds’ representatives	1/2 (50)	1/2 (50)	2/2 (100)		0/2 (0)	0/2 (0)
App certification representative	1/1 (100)	0/1 (0)	1/1 (100)		0/1 (0)	0/1 (0)
Medical chamber representatives	0/4 (0)	4/4 (100)	0/4 (0)		3/4 (75)	1/4 (25)
Political representatives	2/5 (40)	3/5 (60)	0/5 (0)		3/5 (60)	2/5 (40)
Health app producers	2/2 (100)	0/2 (0)	1/2 (50)		1/2 (50)	0/2 (0)

### Ethical Considerations

The ethics approval is not applicable to this study, as we conducted expert interviews. Participants consented the content of the questions. We followed the ESOMAR international code on marketing, opinion, social research, and data analytics [21]. During the expert interviews, we did not ask any personal or confidential content. All questions were subject to health care professional content. All stakeholders agreed in the beginning of the interview to the collection of data and were informed that the pseudonymized transcripts of the interviews are going to be stored at our university server in Germany. No sensitive or personal data were collected.

## Results

Quantitative Results

Within the first coding round, we identified 1048 first-order codes. After discussing their meaning, we merged these codes into 79 first-order codes. Finally, the first-order codes were clustered into 9 second-order codes. These are depicted in Table 2. The interviewee overview and the ATLAS.ti code report, depicting all first- and second-order codes, can be found in the Multimedia Appendix 2.

**Table 2:** Second-order codes. Our calculation was based on ATLAS.ti coding protocol.

Second-order code	Frequency first-order codes (n=79), n	Total number of quotes, n
Factor patient and potential care effects	12	200
Certification process	9	112
Chances for the health care system	7	93
Cost development	7	61
Factor doctor and potential effects on daily routine	14	293
Political incentive systems	8	78
Role of the statutory health insurer and reimbursement	7	58
Considerations for the app developers	5	28
Concerns about data use, data privacy, and data security	10	108

### Chances for the Health Care System

We observed a generally positive perception of the DVG and the option of prescribing health apps as treatments during the interview study. However, most stakeholders would not want to overestimate the effect of health apps introduced as treatments in the German health care system. Medical doctors thought that prescribed health apps should be regarded as optional treatments and not as replacements or substitutions for traditional treatments.

### Factor Patient and Potential Care Effects

The majority of stakeholders thought that the additional option of prescribing health apps could improve treatment quality for patients. The use of health apps has various positive effects for patients, such as more flexibility in terms of location and time as well as a permanent reduction in waiting time for appointments. Most stakeholders argued that the use of health apps could lead to patient emancipation through better disease education and management. It was said that “especially chronic patients could benefit if they need permanent guidance.” However, medical doctors were especially concerned that patients might not use or might incorrectly use the app-based treatment. Therefore, app use supervision and advice from medical doctors should be indispensable. One of the respondents said, “It is important that these technologies are just used with medical supervision, especially for risk patients.” Another concern was that many patients could be excluded from the app treatments because of demographic factors, such as age or local internet connection. One of the respondents said, “An elderly woman aged 70 years—I do not know if she would use these technologies.”

### Factor Doctor and Potential Effects on Daily Routine

Many stakeholders argued that the prescription of DiGA could enhance the service portfolio of resident doctors. One of the respondents said: ”I think that a quality improvement of care is a possible outcome“. Many medical doctors would be delighted if health app use would lead to fewer unnecessary doctor’s visits and therefore again increase treatment time for patients with severe or complicated conditions. Furthermore, medical doctors would have the chance to detect chronic or severe conditions earlier through data insights, which they would not be able to obtain from traditional treatments or patient disease management systems.

### Certification Process

However, the unique certification process might not only be a chance for improvement and innovation but also an opportunity to abuse the system by very high price settings. This could lead to short-term cost increases within the German healthcare system.

### Costs Development

To prevent expensive app collection without use from patients, some stakeholders suggested monitoring compliance and letting patients pay for prescribed apps if they do not use them. On the other hand, statutory-financed health apps also foster the use and perception of health apps in general within society. Technologies such as gamification and nudging may increase patient compliance and use even further for specific treatments.

### Considerations for the App Developers

Some stakeholders recommended a pay-for-performance principle, which means that the final costs for the app should depend on the intensity of the real positive care effect verified during the one-year test phase.

### Political Incentive Systems

There is no sufficiently regulated incentive or remuneration system for physicians who would have an increased workload because of continuous app supervision. Stakeholders from all sectors of the health care system recommended the introduction of individual billing codes and an appealing remuneration system for physicians who supervise app treatments because they fear a blockage of the innovation.

### Concerns About Data Use, Data Privacy, and Data Security

Many stakeholders fear a lack of data security and data privacy for patients; medical doctors especially question the responsibility in cases of data theft and severe personal consequences for patients.

### Role of the Statutory Health Insurer and Reimbursement

All stakeholders recommended that statutory sickness funds, health app producers, and medical chambers in particular should offer a wide portfolio of health app education initiatives to address the needs and interests of physicians with different specialties, ages, location characteristics, and different patient clientele. Therefore, one of the respondents demanded “more education, even workshops about digital treatment solutions because this is important.”

**Figure 1:**
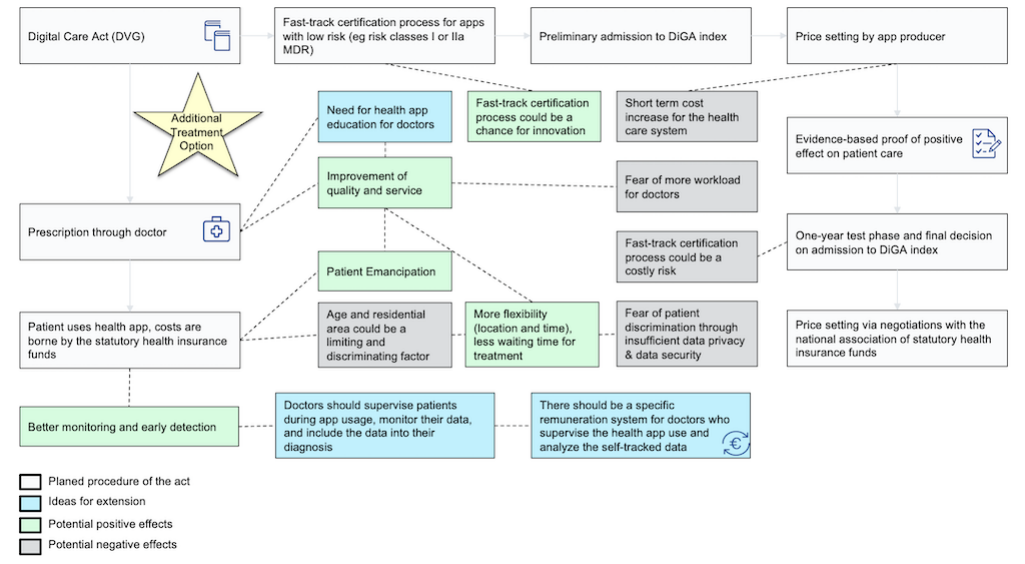
Benefits, risks and recommendations for the Digital Healthcare Act (Digitale Versorgung Gesetz [DVG]). DiGA: Digitale Gesundheitsanwendung (digital health app); MDR: Medical Device Regulation.

Figure 1 presents the main findings and recommendations from the interview study, embedded in the regulatory framework of DiGA certification and implementation.

## Discussion

Mainly, we identified a relative openness toward the introduction of DiGA into standard care. Yet there have been some concerns as well, regarding data security, compensation of medical doctors, and the self-motivation of patients. However, most stakeholders expected benefits resulting from the introduction of the DVG.

One of the major concerns identified during the interview study was that health apps might not provide the desired positive care effects, and therefore, could lead to an unnecessary short-term increase in costs for the German health care system. However, compliance is not just an inhibitor to improvement in the digital sphere but also in the analog treatment world. In particular, medical doctors expressed their concerns in the interview study that digital treatments could lead to a short-term cost increase because the app treatments require self-motivation, which has also been argued by Safi et al [22]. Yet many studies disagree with this standpoint because modern technologies, such as gamification and nudging, have shown a significant positive effect on patient compliance [23,24].

A major advantage of the app treatment versus the traditional treatment is that patients gain location and time flexibility. According to most stakeholders, this advancement could lead to an improvement in treatment quality and service due to an extension of health care portfolios. Dahlhausen et al [25] came to similar conclusions resulting from their survey about DiGA with German practitioners.

All stakeholders agreed that there is a need to introduce an appealing and individual financial incentive system to remunerate the increased workload that medical practitioners have due to continuous app advice, supervision, and data analysis. All stakeholders proposed individual billing codes for practitioners based on workload increase to ensure the support and participation of these important gatekeepers.

The opportunities that app treatments offer through data generation and patient monitoring could improve research and diagnostics to a large extent because of their regular real-world and real behavioral documentation [26]. App treatments are not supposed to replace traditional treatments, but app-based treatments offer many opportunities and additional benefits, which is why app-based treatments should be regarded as a valuable complement to medical care portfolios [25]. Yet a representative survey with practitioners showed that 33.6% of the participating physicians have already prescribed a DiGA in 2022 [27]. In 2021, just 14.3% prescribed DiGA; and in 2020, just 1% did so [27]. This means we see a fast adoption rate, and contrary to our hypothesis, a general openness to prescribe and use DiGA in the standard care setting.

The educational elements of the apps might additionally lead to more patient emancipation through a better understanding of personal conditions. Location and time flexibility are the biggest advantages of the new technologies, but they also raise the most significant concerns for stakeholders because app use requires personal initiative and self-motivation, as also argued by Weise et al [28]. Physicians should supervise and monitor patients’ app use to support the adequate use of the app as a treatment. This supervision might also help to prevent patients from collecting but not using reimbursable health apps, and therefore, exploiting the system.

We conducted this study prior to the COVID-19 crisis. Hence, we now expect an increased positive perception of DiGA due to experiences during the lockdown in Germany introduced on March 25, 2020.

In conclusion, stakeholders within the German health care system had generally an open mind toward the Digital Healthcare Act and felt that the introduction of the act helps to relieve the dust from the German health care system and pushes forward the digitization of the industry. The inclusion of health apps in the statutory health care system could improve the quality and service of treatment by expanding portfolios, and therefore, is considered beneficial by most stakeholders.

## Appendix

Multimedia Appendix 1 Interview questionnaire guide.

Multimedia Appendix 2 Interview partner overview and code report.

## Abbreviations

BfArM: Bundesinstitut für Arzneimittel und Medizinprodukte (Federal Institute for Drugs and Medical Devices)
DiGA: Digitale Gesundheitsanwendung (digital health app)
DiGAV: Digitale-Gesundheitsanwendungen-Verordnung (digital device regulation)
DVG: Digitale Versorgung Gesetz (Digital Healthcare Act)
GOP: Gebührenordnungsposition (billing code)
SGB: Sozialgesetzbuch (German social code)
