# Supplementary material for: Removing Dust From the German Health Care System by Introducing Health Apps Into Standard Care: Semistructured Interview Study
Source: JMIR Hum Factors. 2023 May 4;10:e42186. doi: 10.2196/42186 (PMC10196889; doi:10.2196/42186)
Supplement: Multimedia Appendix 1 [file humanfactors_v10i1e42186_app1.docx]

**Multi-Media Appendix 1 - Interview Questionnaire Guide**

1. **Beginning of the interview:**
2. Brief introduction to the topic and possibly a brief summary of Digital Care Act (DVG).
3. Explain the basic functionalities of health apps.
4. Ask for Participant & Data Processing Consent – inform about the recording of the interview
5. **General part:**
6. In your opinion, what effects will the Digital Care Act (DVG) have?
7. What advantages and disadvantages do you generally see for the German healthcare system through greater use of mHealth?
8. Do you think that the draft law can contribute to increasing the quality of treatment? If yes, in which aspects exactly? If not, what are the reasons why you think this is not the case?
9. What costs or cost savings do you could be caused by the draft law?
10. **Detailed questions about the DVG:**
11. What do you think about the approach that statutory health insurance companies cover the costs of DiGA according to DVG?
12. How do you think about the fee cuts planned as part of the draft law for medical doctors who do not participate in digital patient care?
13. What problems and obstacles do you see in accordance with the implementation of the act? What consequences could this have exactly?
14. What would you like to change about the act?
15. **Areas of application:**
16. In which areas of healthcare do you see the greatest potential for mHealth?
17. In your opinion, what could be the reason for differences in the use of mHealth between general practitioners and specialists?
18. In which areas in particular do you see the possibility that routine work can be reduced?
19. In your opinion, could mHealth offer new types of treatment that increase the quality of treatment?
20. What specific type of mHealth products should physicians prescribe?
21. **Questions about the individual stakeholders in the healthcare system:**
22. Do you think that greater integration of mHealth products can help physicians spend their time more efficiently on treatment?
23. Do you think patients will use health apps as a substitute for doctor visits? To what extent would this be assessed positively / negatively?
24. Do you think that physicians could improve diagnostic and educational aspects by prescribing mHealth products in their daily practice and hospital routine? If yes, how exactly?
25. What additional burdens do you see for doctors due to the evaluation of mHealth data or possible incorrect diagnoses?
26. Do you think patients will embrace the use of health apps as an option? If not, what doubts do you have exactly?
27. Do you see ways in which health insurance companies can use health apps to improve their service?
28. What cost savings or what burdens do you see for health insurance companies that could result from the planned regulations?
29. **Specific part (depending on the interviewee):**

Doctors:

1. Are you already using mHealth products?
2. If so, which products do you use, in which area do you use them and what was the feedback from your patients on the use of mHealth products?
3. If so, can you already improve the quality of your medical advice or save costs with mHealth products?
4. Have you ever had to stop using a particular mHealth product in the past? If yes, what were the reasons?
5. Which chances and which disadvantages could arise specifically for you from the legislative proposal?
6. Do you think that you have been able to improve the quality of the medical care you offer by using mHealth?
7. How might integrating mHealth products change how you treat patients? Could these changes improve patient well-being?
8. What specific costs would the proposed legislation entail for you? Could you possibly save costs with the legislative proposal?

Statutory health insurance companies:

1. Are you already using mHealth products?
2. What advantages and disadvantages could result from the draft law for your insured?
3. What advantages and disadvantages could result from the draft law for you as a health insurance company?
