# Supplementary material for: Removing Dust From the German Health Care System by Introducing Health Apps Into Standard Care: Semistructured Interview Study
Source: JMIR Hum Factors. 2023 May 4;10:e42186. doi: 10.2196/42186 (PMC10196889; doi:10.2196/42186)
Supplement: Multimedia Appendix 2 [file humanfactors_v10i1e42186_app2.docx]

**Multimedia Appendix 2**

1. **Interview Partner Overview**

| Nr. | Interview Partner | Date | Gender | Age Group | Participant & Data Processing Consent |
| --- | --- | --- | --- | --- | --- |
| 1 | Physician 1 | 07. Oct 2019 | female | III | Yes |
| 2 | Physician 2 | 07. Oct 2019 | male | I | Yes |
| 3 | Physician 3 | 20. Nov 2019 | female | II | Yes |
| 4 | Physician 4 | 22. Nov 2019 | female | III | Yes |
| 5 | Physician 5 | 22. Nov 2019 | male | III | Yes |
| 6 | Physician 6 | 26. Nov 2019 | female | II | Yes |
| 7 | Physician 7 | 27. Nov 2019 | male | II | Yes |
| 8 | Physician 8 | 28. Nov 2019 | male | III | Yes |
| 9 | Physician 9 | 28. Nov 2019 | female | II | Yes |
| 10 | Physician 10 | 29. Nov 2019 | male | I | Yes |
| 11 | Statutory Health Insurance Representatives 1 | 14. Oct 2019 | male | II | Yes |
| 12 | Statutory Health Insurance Representatives 2 | 30. Oct 2019 | female | II | Yes |
| 13 | Medical Chamber Representatives 1 | 1. Oct 2019 | male | II | Yes |
| 14 | Medical Chamber Representatives 2 | 31. Oct 2019 | male | III | Yes |
| 15 | Medical Chamber Representatives 3 | 6. Nov 2019 | male | II | Yes |
| 16 | Medical Chamber Representatives 4 | 11. Nov 2019 | male | II | Yes |
| 17 | Health App producers or App Certification Representative 1 | 01. Nov 2019 | female | II | Yes |
| 18 | Health App producers or App Certification Representative 2 | 07. Nov 2019 | male | II | Yes |
| 19 | Health App producers or App Certification Representative 3 | 19. Nov 2019 | male | I | Yes |
| 20 | Political Representatives 1 | 28. Oct 2019 | male | II | Yes |
| 21 | Political Representatives 2 | 08. Nov 2019 | male | II | Yes |
| 22 | Political Representatives 3 | 04. Dec 2019 | female | III | Yes |
| 23 | Political Representatives 4 | 12. Dec 2019 | female | III | Yes |
| 24 | Political Representatives 5 | 07. Jan 2020 | male | II | Yes |

Group I: < 35

Group II. 35 - 50

Group III: > 50

1. **Code Report – Interview Study**

| **Title** | ATLAS.ti – Code-Report |
| --- | --- |
| **Project:** | Digital Healthcare Act |
| **Date:** | 07.05.2020 - 14:40:32 |

| **Code-Group** | **Code** | **Frequency** |
| --- | --- | --- |
| Certification Process | Apps need to be certified as medical devices (EU law) | 8 |
|  | BfArM good agent for certification process | 8 |
|  | BfArM not right agent to certify apps - need for new interdisciplinary board | 6 |
|  | catalogue of criteria | 4 |
|  | certification process too fast - proof of effectiveness before certification | 26 |
|  | evidence testing should be different to costly medication testing | 9 |
|  | Great chance to test a new treatment for 12 months - fast track | 25 |
|  | need for more experience - learning by implementing | 17 |
|  | risk, evidence could be too low but yet certified | 9 |
| Chances for the Health Care System | better disease management, education & monitoring | 21 |
|  | Early Detection of Conditions | 9 |
|  | flexibility through time and waiting time reduction | 4 |
|  | increase of treatment quality | 35 |
|  | individualized therapy | 2 |
|  | Skepticism about ability of health apps and the improvement of treatment quality | 13 |
|  | service improvement for patient | 9 |
| Concerns about Data Use, Data Privacy and Data Security | Data analysis through health insurer | 3 |
|  | data as advantage for doctor’s diagnosis and therapy | 13 |
|  | data could be very valuable for research | 18 |
|  | data privacy and data security manageable, no big problem | 5 |
|  | data privacy sometimes blocks innovation | 4 |
|  | Data Sales to Third Parties and Discrimination | 12 |
|  | data security and data privacy very important and should have highest priority | 18 |
|  | government should protect and regulate data storage and usage | 17 |
|  | patient should be owner of data | 13 |
|  | personal data should be anonymized, transparency about processing | 5 |
| Considerations for the App Developer | app producer should educate doctors | 4 |
|  | apps need proof of effectiveness | 5 |
|  | high market entry barriers | 3 |
|  | Potential for free rider problem | 6 |
|  | risk price setting | 10 |
| Cost Development | Costs depend on demand | 3 |
|  | initial increase in costs | 25 |
|  | more efficiency | 5 |
|  | not very high increase of costs | 3 |
|  | Potential cost reduction in the long run, depends on app | 12 |
|  | Prevention saves indirect costs | 4 |
|  | Skepticism about cost reduction through digitization and the efficiency of health apps | 9 |
| Factor Doctor and Potential Effects on Daily Routine | age, demographics and personal interests of doctors play a role | 15 |
|  | all actors of health care sector will benefit (specialists and generalists) | 23 |
|  | apps could reduce unnecessary doctors’ visits | 24 |
|  | Apps do not reduce doctors’ visits | 6 |
|  | could help to counter lack of physicians in rural areas, decrease ways to doctors for patients | 3 |
|  | doctor plays main role | 8 |
|  | Doctors are skeptical | 36 |
|  | doctors have little knowledge about apps | 42 |
|  | doctors need health app education, many are already left behind | 36 |
|  | doctors need positive monetary incentive to prescribe and supervise health app use | 20 |
|  | Doctors Responsibility | 8 |
|  | doctors should monitor app usage | 23 |
|  | Increase in quality through better diagnostics, early detection and monitoring | 24 |
|  | increase of workload | 25 |
| Factor Patient and potential App Effects | accessibility | 2 |
|  | Age & demographic factors are no excluding criteria | 23 |
|  | age, demographics and digital literacy/interest as limiting factor | 62 |
|  | Chances to increase adherence & compliance and change patient behavior | 32 |
|  | High patient demand | 8 |
|  | increase in patient education, disease management and patient emancipation | 32 |
|  | Little demand | 5 |
|  | no mobility & time constraints - flexibility | 6 |
|  | Payment scenario should be different | 2 |
|  | Risk of unguided self-assessment and misinterpretation | 12 |
|  | trust | 6 |
|  | Usability and technology literacy | 10 |
| Political Incentive Systems | DVG enables digitization and innovation in health care sector | 19 |
|  | Germany very special with data security | 2 |
|  | Inclusion of doctors in decision making introduction of reward system | 9 |
|  | negative constraints as motivation obstacle triggers rejection | 16 |
|  | Negative constraints as useful and necessary instrument | 14 |
|  | pilot character for other countries | 4 |
|  | regulation shell trigger digitization in health care sector - investment gap | 7 |
|  | technical compatibility between systems | 7 |
| Role of the Statutory Health Insurer and Reimbursement | Apps should be payed or co-payed by patients | 12 |
|  | health insurer should not be health app provider | 6 |
|  | Incentive based health insurance plan | 2 |
|  | more active role for health insurer, quality improvement | 10 |
|  | selective treaties and negotiation with health App provider plausible | 5 |
|  | statutory health insurance should pay, good incentive system | 18 |
|  | threat for solidarity-based system | 5 |
